# Left ventricular free-wall rupture, a potentially lethal mechanical complication of acute myocardial infarction: an unusual and illustrative case report

**DOI:** 10.1186/s12872-019-1063-x

**Published:** 2019-04-03

**Authors:** Dulman O. Pineda-De Paz, Jorge E. Hernández-del Rio, Christian González-Padilla, Ramón M. Esturau-Santaló, Joseph Romero-Palafox, Fernando Grover-Paez, David Cardona-Muller

**Affiliations:** 1 Departamento de Cardiología, Antiguo Hospital Civil de Guadalajara “Fray Antonio Alcalde”, Centro Universitario de Ciencias de la Salud, Universidad de Guadalajara, Hospital 278, Col. El Retiro, 44280 Guadalajara, Jalisco México; 2 Centro Universitario de Ciencias de la Salud, Universidad de Guadalajara, Sierra Mojada 950, Col. Independencia, Guadalajara, Jalisco México; 3 Departamento de Fisiología, Centro Universitario de Ciencias de la Salud, Universidad de Guadalajara, Sierra Mojada 950, Col. Independencia, Guadalajara, Jalisco México

**Keywords:** Left ventricle, Pseudoaneurysm, Myocardial infarction, Case report

## Abstract

**Background:**

There are three major mechanical complications after acute myocardial infarction: left ventricular free-wall rupture, ventricular septum rupture and acute mitral valve regurgitation. The left ventricular free-wall rupture is a serious and often lethal complication following an ST elevation myocardial infarction. However, very rarely this rupture can be contained by the pericardium, forming a pseudoaneurysm.

**Case presentation:**

We report a case of a 66-year-old man with multiple cardiovascular risk factors and previous ST elevation myocardial infarction, complaining of atypical chest pain. His electrocardiogram was in normal sinus rhythm, with the presence of Q wave in inferior leads and T-wave inversion in lateral leads. A transthoracic echocardiogram showed a left ventricular pseudoaneurysm. In the coronary angiography, multi-vessel disease was found. On-pump CABG was performed and a posterolateral left ventricular giant pseudoaneurysm were observed. Due its “petrous” consistency it was impossible to perform an aneurysmectomy.

**Conclusions:**

The diagnosis of left ventricular pseudoaneurysm can be difficult, as patients often present either asymptomatic or with non-specific symptoms attributed to other causes. A multimodality imaging diagnostic approach can be necessary. Immediate surgery is considered the treatment of choice because untreated pseudoaneurysms have a high risk of rupture leading to cardiac tamponade, shock and death.

## Background

There are three major mechanical complications after acute myocardial infarction: left ventricular (LV) free-wall rupture, interventricular septum rupture, and acute mitral valve regurgitation [1]. Acute or subacute LV free-wall rupture is a “serious” and often “lethal” complication of ST elevation myocardial infarction (STEMI), reported as a common finding in patients who die from acute myocardial infarction (MI) [1, 2].

These are rare complications [3]. LV free-wall rupture after MI has an incidence of 2–4% [3]. Considering all patients who suffer acute MI, the incidence of LV pseudoaneurysm (PSA) is low (< 1%) and even lower when the patients undergo primary percutaneous coronary intervention (PCI), considering the latter as a protective factor [3].

## Case presentation

A 66-year-old man with multiple cardiovascular risk factors; such as diabetes, hypertension, smoking and STEMI 6 years ago, who did not receive a reperfusion therapy. He consulted the ER referring atypical chest pain that began 8 days prior to his visit. He showed normal vital signs with the following relevant findings in the cardiovascular physical examination: visible and palpable double systolic apical impulse with a wide apical impulse area (4,5 cm in diameter), located in the fifth intercostal space of the left mid-clavicular line. On auscultation, an audible fourth heart sound (S4) was present. The cardiac biomarkers were negative.

### 12-Lead rest ECG

**Fig. 1:**
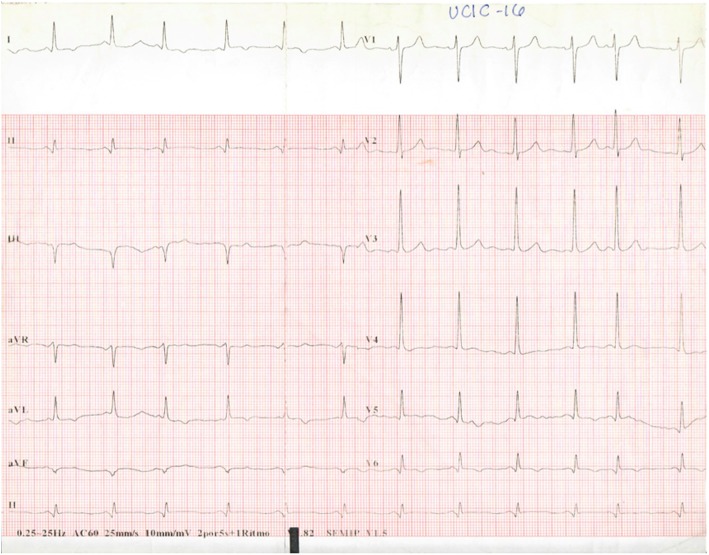
12-Lead rest ECG showing normal sinus rhythm, Q-wave in inferior leads and asymmetrical T-wave inversion in lateral leads

Normal sinus rhythm, Q-wave in inferior leads and T-wave inversion in lateral leads. (Fig. 1).

### Chest x-ray

**Fig. 2:**
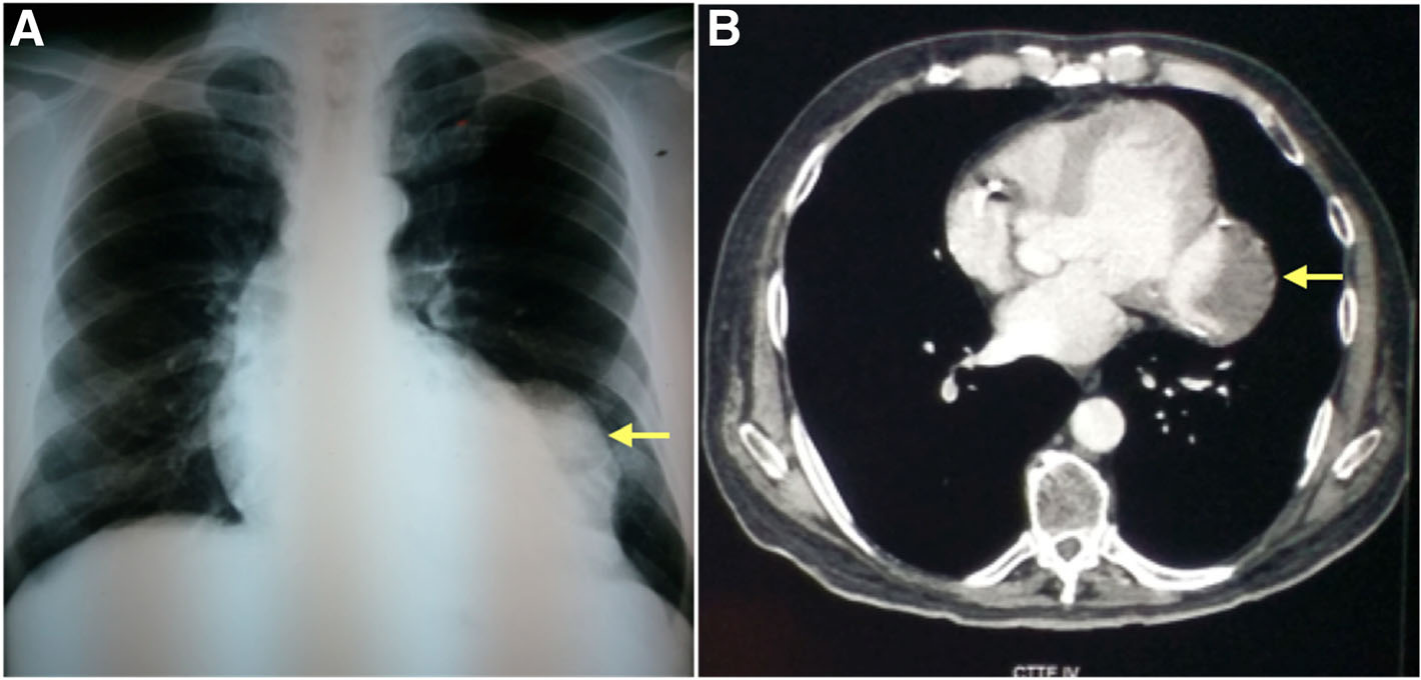
**a** Chest x-ray showing mild cardiomegaly, of note, a homogeneous opacity adjacent to the LV (arrow). **b** Chest CT showing a saccular image in the posterolateral wall of the LV that corresponds to a thrombosed PSA in the LV free -wall (arrow)

Mild cardiomegaly, of note, a homogeneous opacity was observed adjacent to the LV. (Fig. 2a).

### Chest CT

Revealed a saccular image in the LV posterolateral wall, which, due to its characteristics, suggested a thrombosed PSA in the LV free -wall. (Fig. 2b).

### Transthoracic echocardiogram (TTE)

**Fig. 3:**
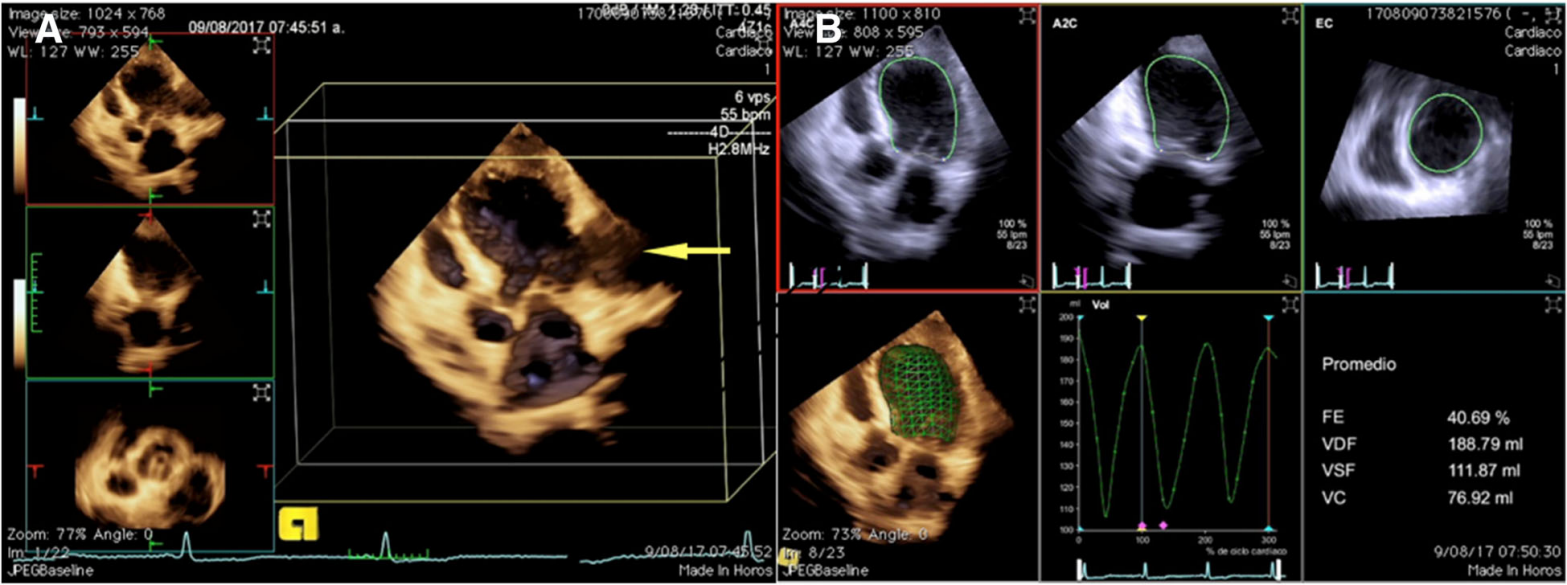
3D transthoracic echocardiogram showing a dilated left ventricular cavity and the PSA [arrow] (**a**) and the calculation of the LV end diastolic and systolic volumes, and LV ejection fraction (40%) by 3D method (**b**)

**Fig. 4:**
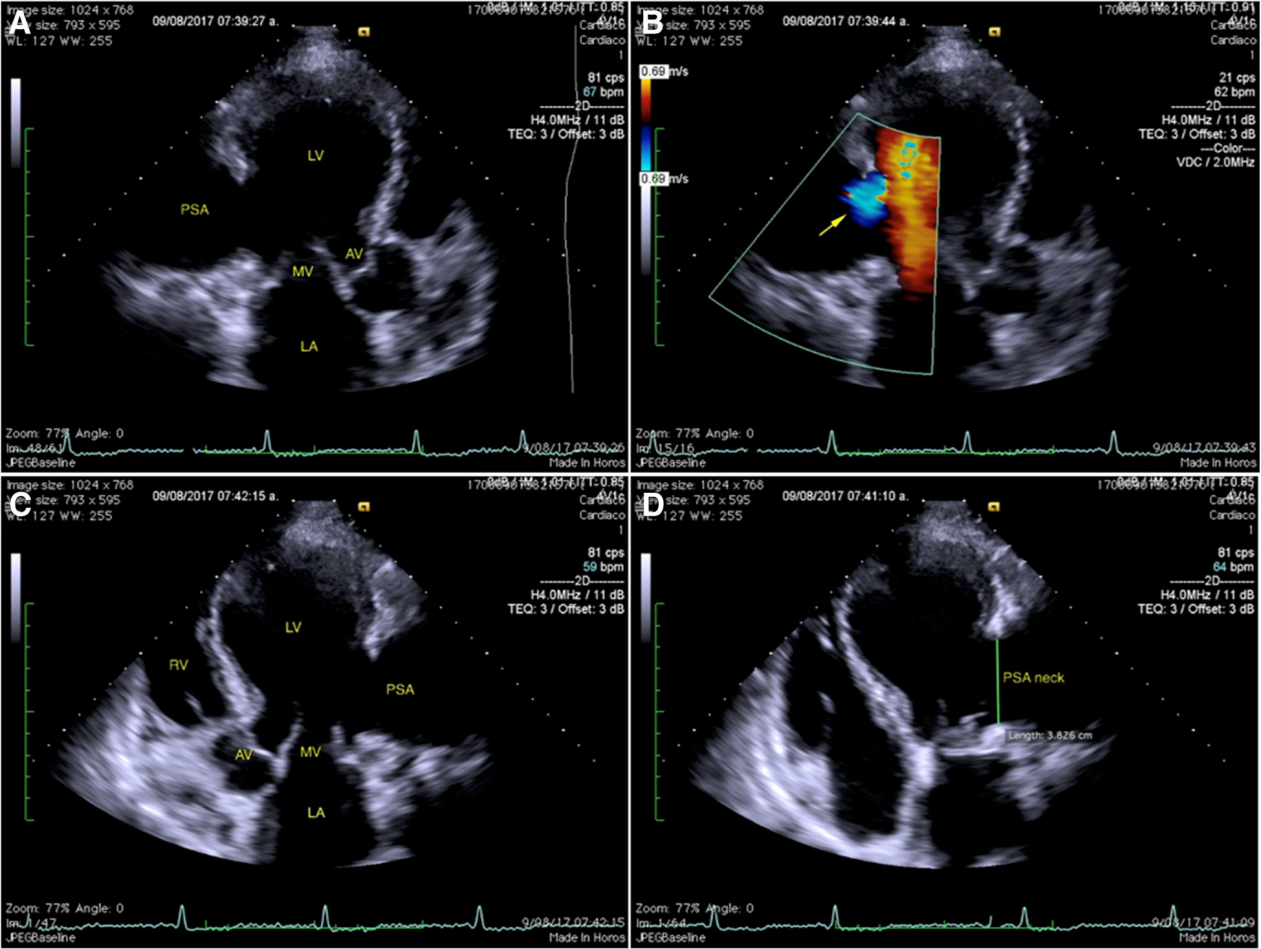
2D transthoracic echocardiogram: **a** Apical 3-chamber view, showing a spherical-shaped left ventricular cavity and a PSA involving the basal and mid segments of the inferolateral wall. **b** Apical 3-chamber view with colour Doppler showing a shunt from the LV to PSA (arrow). **c** Apical 5-chamber view showing a PSA involving the basal and mid segments of the anterolateral wall. **d** Apical 4-chamber view showing a PSA involving the basal and mid segments of the anterolateral wall with a narrow neck (38 mm)

Showed a spherical-shaped left ventricular cavity with segmental wall-motion abnormalities, a LV ejection fraction of 40% by 3D method, (Fig. 3) PSA involving the basal and mid segments of both, inferolateral and anterolateral wall; with a narrow neck (38 mm), a shunt of LV to PSA was observed in color Doppler. (Fig. 4).

### Myocardial perfusion imaging (thallium^201^) by SPECT

**Fig. 5:**
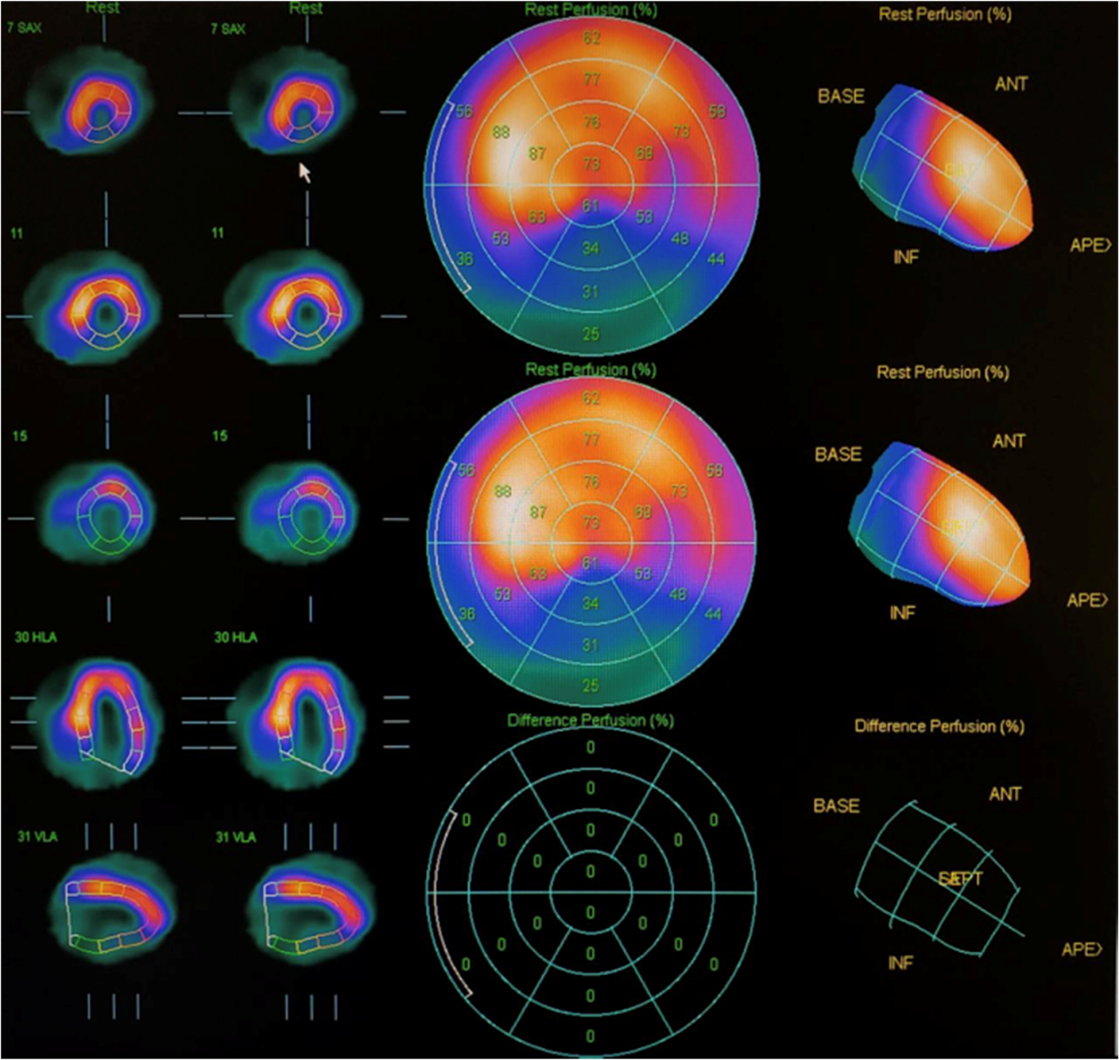
Myocardial perfusion imaging (thallium^201^) by SPECT: showing a myocardial infarction located in inferolateral region, which involved the inferoseptal region; a non-transmural infarction in the apical segment, and transmural in basal and mid segment, without showing viability in delayed redistribution imaging

A viability protocol rest imaging/4-h redistribution imaging/24-h redistribution imaging was performed and showed a myocardial infarction located in the inferolateral wall, which involved the inferoseptal region; non-transmural in the apical segment and transmural in basal and mid segment, without signs of viability in the delayed redistribution imaging. (Fig. 5).

### Coronary angiography

**Fig. 6:**
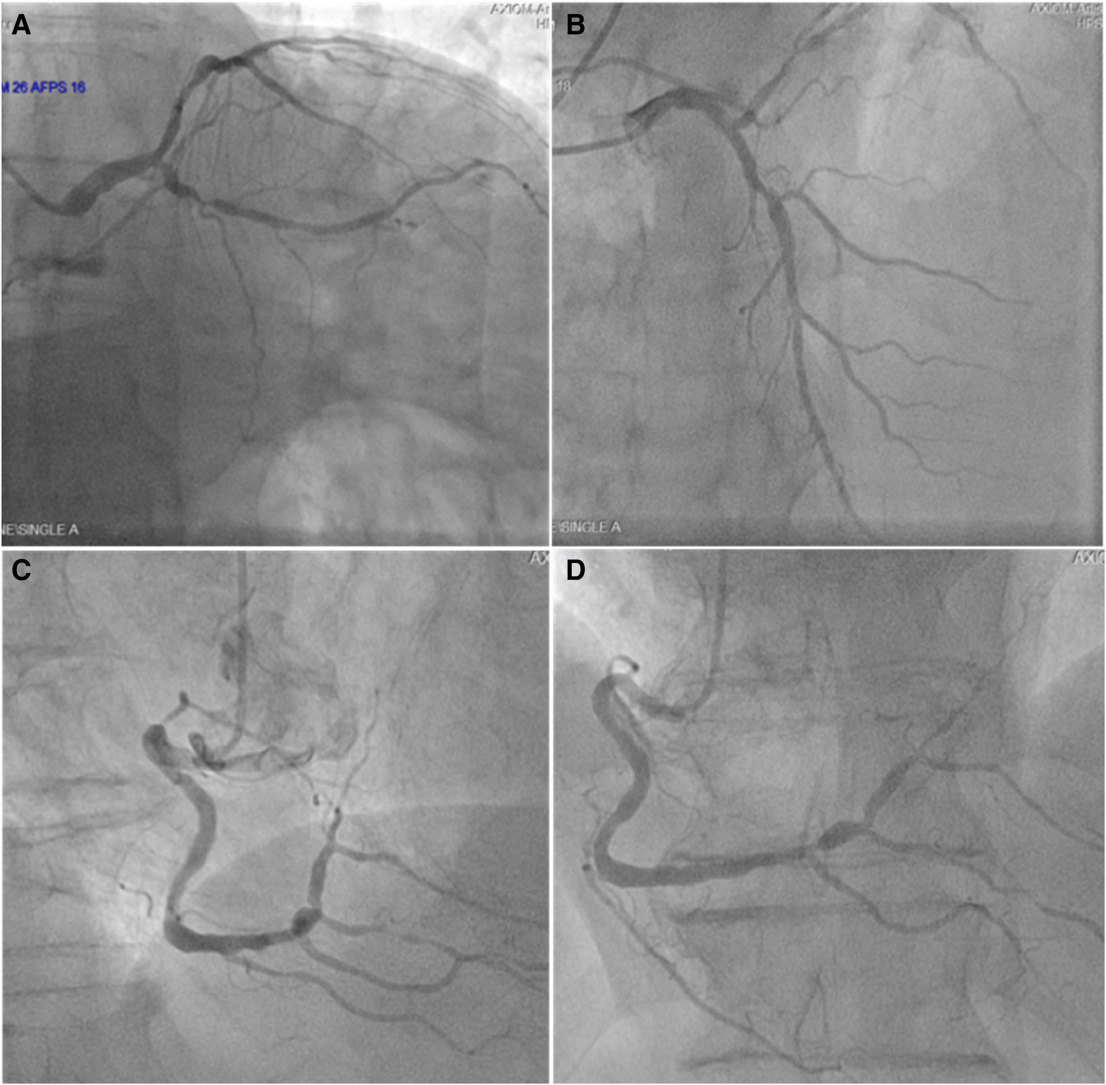
Coronary Angiography: **a** Left coronary angiogram. RAO caudal projection showing a significant LM stenosis, diffuse atherosclerotic disease with significant obstruction of de LCX is also apparent. **b** AP/Cranial projection showing significant LAD proximal and mid segment atherosclerotic disease. **c** and **d** Right coronary angiogram. Significant atherosclerotic obstruction in the distal segment of the RCA, which involves both; posterolateral and PD branches

Multi-vessel coronary artery disease, with involvement of the left main coronary artery and high SYNTAX score [40 pts.]. (Fig. 6) Left ventriculography was not performed due to elevated end diastolic pressure and because of the previously reported left ventricular mural thrombus.

The case was discussed by the heart team. Due to the high SINTAX score and the mechanical complication, a coronary artery bypass grafting (CABG) plus aneurysmectomy with geometric reconstruction was considered. On-pump CABG was performed with internal thoracic artery anastomosis to the left anterior descendent coronary artery. A giant posterolateral wall LV PSA of approximately 7 cm was observed; described with a “petrous” consistency and adhered to the posterior pericardium. Due to the previously mentioned findings, it was not possible to perform the aneurysmectomy, as the cardiovascular surgical team considered the benefit was not worth compared to the risk of performing the procedure, so the surgery was concluded. The patient was discharged five days after CABG and he is currently asymptomatic, in NYHA functional class II and is followed up as an outpatient in our hospital.

## Discussion and conclusions

We discuss the case of a 66-year-old male with a history of MI, six-years prior to consulting our hospital; that due to lack of access to a cath lab, the patient didn’t receive any reperfusion strategy. Thus, the patient only received medical treatment as an inpatient in a 2nd level hospital and the initial symptoms associated to the pseudoaneurysm were probably considered as part of the symptoms related to an acute MI.

Myocardial rupture usually occurs within the first 3–5 days after the infarction in half of the cases (early rupture) and in the first two weeks in 90% (late rupture) [4]. The clinical course varies, being one of the causes of sudden death in asymptomatic acute MI [4, 5].

The LV free-wall rupture can be complete or incomplete [6]. Complete rupture triggers hemopericardium and sudden death from cardiac tamponade, whereas incomplete rupture may occur when an organized thrombus and the pericardium seals the ventricular perforation forming a ventricular PSA [6].

LV PSA is a rare disorder but of great clinical relevance due to its high associated mortality [7]. It usually occurs after transmural myocardial infarction, being the most common cause of LV PSA [7]. They typically have a narrow neck, lacking the elements of the original ventricular wall (myocardium and endocardium) and are more often located in the posterior and lateral wall segments, in contrast with true aneurysms which are more often seen in the anterior wall and apex, they always contain myocardium in their wall and have a wide neck [7, 8].

The diagnosis of LV PSA can be difficult because patients are often either asymptomatic, or present with non-specific symptoms attributable to other causes. It can even be diagnosed incidentally. A multimodality imaging diagnostic approach can be necessary, including transthoracic/transoesophageal echocardiogram, cardiovascular magnetic resonance (CMR), chest CT, myocardial perfusion imaging (MPI) and LV angiography [4, 9].

European Society of Cardiology guidelines for the management of STEMI establish that the diagnosis is confirmed with a TTE. These guidelines consider CMR can complement the diagnosis by identifying the contained cardiac rupture and its anatomical features to guide surgical interventions [10]. In this case report, we confirmed the diagnosis with TTE and obtained complementary information with other imaging modalities like Chest CT scan and MPI. We considered the information obtained with these techniques sufficient to establish the diagnosis and to guide the surgical intervention.

Immediate surgery is the treatment of choice, because an untreated PSA has a 30 to 45% rupture risk [11]. The patient underwent surgery with a programed two-step intervention, aneurysmectomy plus CABG, as is recommended in the clinical practice guidelines [10]. In the setting of a PSA, aneurysmectomy with a pericardial patch (or other material) is recommended [10]. The mortality rates in this type of interventions are in the order of 20–75% [10]. The morphologic characteristics encountered in this particular PSA suggested chronicity; we believe that this PSA could have occurred years before the diagnosis at our hospital, hence the “petrous” consistency which made resection impossible. Regarding the surgical treatment received by the patient, there are to our knowledge no available reports that establish a recommendation with respect to a specific treatment of choice in the setting of a giant PSA with “petrous” consistency. Due to the patient’s current clinical condition, we believe that the correct treatment option was chosen.

The present case report is interesting because it is unusual to find this mechanical complication of MI. Its low incidence is probably secondary to a greater accessibility to primary PCI (which has been previously described as a protective factor). This patient didn’t receive any reperfusion therapy (fibrinolysis/PCI), before attending our Centre; therefore, he was more likely to present this potentially fatal complication. In addition, the multimodality cardiac imaging used in this case, is very illustrative of this MI “serious” mechanical complication.

When encountered, PSA requires immediate surgery because an untreated LV PSA has a high risk of rupture, leading to cardiac tamponade, shock and death. CABG plus aneurysmectomy with geometric reconstruction was considered for this patient following guideline recommendations; however in this case, only successful CABG was performed; the “petrous” consistency of the PSA made aneurysmectomy impossible to perform.

Finally, although a high mortality without surgery is reported, our patient maintains a favorable evolution two-years post CABG; he is currently in NYHA II functional class, receiving optimal medical treatment and regular follow-up visits as an outpatient in our Centre.

In conclusion, the LV free-wall rupture is a serious and often lethal complication of STEMI resulting a common finding in patients who die from acute MI. LV PSA is a rare disorder but of great clinical relevance due to the high associated mortality. The diagnosis of LV PSA can be difficult and often requires a multimodality imaging diagnostic approach. This MI “serious” mechanical complication can be prevented by primary PCI, however, when it is found, warrants surgical treatment. This particular case is atypical. The long evolution of this complication made surgical resection impossible, despite this setback, the patients’ evolution was favorable.

## Abbreviations

CABG: Coronary artery bypass graft
ECG: Electrocardiogram
LV: Left ventricle
MI: Myocardial infarction
NYHA: New York Heart Association
PSA: Pseudoaneurysm
SPECT: Single-photon emission computed tomography
STEMI: ST elevation myocardial infarction
TTE: Trans-thoracic echocardiogram

## References

[1] Khalid S, Seepana J, Sundhu M, et al. Left Ventricular Free Wall Rupture in Transmural Myocardial Infarction. Cureus. 2017;9(8):e1610. 10.7759/cureus.1610. 10.7759/cureus.1610 PMC5656223 29075588

[2] Masuda S, Shibui T, Onodera R, Ashikaga T. A case of left ventricular pseudoaneurysm presenting with a visible apex beat. Eur Heart J - Case Rep. 2018;2(Issue 2):yty052. 10.1093/ehjcr/yty052. 10.1093/ehjcr/yty052 PMC6177029 31020131

[3] Moreno R, López-Sendón J, García E, De Isla LP, De Sá EL, Ortega A (2002). Primary angioplasty reduces the risk of left ventricular free wall rupture compared with thrombolysis in patients with acute myocardial infarction. J Am Coll Cardiol.

[4] Marchandot B, Crimizade U, El GS, Morel O (2018). Giant ventricular pseudoaneurysm following inferior myocardial infarction : insights from multimodal imaging approach. Eur Heart J.

[5] Ludmir J, Kapoor K, George P, Khural J, Barr B (2016). Left Ventricular Pseudoaneurysm Following Inferior Myocardial Infarction: A Case for Conservative Management. Cardiol Res.

[6] Frances C, Romero A, Grady D. Left Ventricular Pseudoaneurysm. J Am Coll Cardiol; 1998;32(3):557–561. Available from: 10.1016/S0735-1097(98)00290-3. Elsevier Masson SAS 10.1016/s0735-1097(98)00290-3 9741493

[7] Prifti E, Bonacchi M, Baboci A, Giunti G, Veshti A, Demiraj A, et al. Surgical treatment of post-infarction left ventricular pseudoaneurysm : Case series highlighting various surgical strategies. Ann Med Surg. 2017;16:44–51. Available from: 10.1016/j.amsu.2017.03.013. Elsevier Ltd 10.1016/j.amsu.2017.03.013 PMC5369265 28386394

[8] Bisoyi S, Dash AK, Nayak D, Sahoo S, Mohapatra R (2016). Left ventricular pseudoaneurysm versus aneurysm a diagnosis dilemma. Ann Card Anaesth.

[9] Tuan J, Kaivani F, Fewins H (2008). Left ventricular pseudoaneurysm. Eur J Echocardiogr.

[10] Ibanez B, James S, Agewall S, Antunes MJ, Bucciarelli-Ducci C, Bueno H, et al. 2017 ESC guidelines for the management of acute myocardial infarction in patients presenting with ST-segment elevation. Eur Heart J [Internet] 2018;39(2):119–177. Available from: 10.1093/eurheartj/ehx637 10.1016/j.rec.2017.11.010 29198432

[11] Soud M, Moussa H, Hritani R, Alraies MC (2018). Cardiovascular Revascularization Medicine Post myocardial infarction left ventricular pseudoaneurysm. Cardiovasc Revascularization Med.

